# Experimental verification and identifying biomarkers related to insomnia

**DOI:** 10.3389/fneur.2023.1189076

**Published:** 2023-11-28

**Authors:** Qianfei Wang, Dong Liu, Tianci Gao, Yulei Tao, Xin Li, Yuan Liu, Zhiliang Liu, Jianqiang Mei, Fenqiao Chen

**Affiliations:** ^1^The Graduate School, Hebei University of Chinese Medicine, Shijiazhuang, China; ^2^The First Affiliated Hospital, Hebei University of Chinese Medicine, Shijiazhuang, China; ^3^The Emergency Department, Hebei Yiling Hospital, Shijiazhuang, China

**Keywords:** insomnia, sleep deprivation, gene, pathways, RNA, immune infiltration

## Abstract

**Introduction:**

Insomnia is the most common form of sleep deprivation (SD) observed in clinics. Although there are differences between insomnia and SD, they have similar symptoms and the same animal model. Currently, there is a lack of microarray data on insomnia. Therefore, for now, we are going to apply the SD data to insomnia. Although many studies have explained the possible mechanisms associated with insomnia, no previous studies have considered the key genes associated with insomnia or the relationship between insomnia and immune cells. In this study, we analyzed the relationship between key genes and immune cells by identifying biomarkers for the diagnosis of insomnia. Next, we verified the efficacy of these biomarkers experimentally.

**Methods:**

First, we downloaded four microarrays (GSE11755, GSE12624, GSE28750, and GSE48080) from the Gene Expression Omnibus (GEO) database, which included data from 239 normal human blood samples and 365 blood specimens from patients with SD. Then, we analyzed two groups of differentially expressed genes (DEGs) and used Support Vector Machine Recursive Feature Elimination (SVM-RFE) analysis and the Least Absolute Shrinkage and Selection Operator (LASSO) regression model to investigate these key genes. Next, we used CIBERSORT to investigate the composition of 22 immune cell components of key genes in SD patients. Finally, the expression levels of key biomarkers in sleep-deprived patients were examined by quantitative real-time polymerase chain reaction (qRT-PCR).

**Results:**

A total of 50 DEGs were identified: six genes were significantly upregulated, and 44 genes were significantly downregulated. Kyoto Encyclopedia of Genes and Genomes (KEGG) pathway analysis showed that Salmonella infection, NOD-like receptor (NLR) signaling pathway, Kaposi sarcoma-associated herpesvirus infection, and Th17 cell differentiation were significant. Based on machine learning, we identified *C2CD2L, SPINT2, APOL3, PKNOX1*, and *A2M* as key genes for SD; these were confirmed by receiver operating characteristic (ROC) analysis. Immune cell infiltration analysis showed that *C2CD2L, SPINT2, APOL3, PKNOX1*, and *A2M* were related in different degrees to regulatory T cells (Tregs), follicular T helper cells, CD8 cells, and other immune cells. The qRT-PCR experiments confirmed that the expression levels of *C2CD2L* concurred with the results derived from machine learning, but *PKNOX1* and *APOL3* did not.

**Discussion:**

In summary, we identified a key gene (*C2CD2L*) that may facilitate the development of biomarkers for insomnia.

## 1 Introduction

Sleep deprivation (SD) is a form of sleep disorder that does not allow individuals to conform to a normal sleep pattern (1, 2). Long-term SD can lead to schizophrenia, a condition that can seriously affect a patient's quality of life (3, 4). Insomnia is the most common form of SD observed in clinics and is caused by illness or emotional factors without any compulsive factors (5). Long-term insomnia can also lead to a decline in a patient's quality of life. SD and insomnia have some similarities. Patients with these sleep disorders lack good quality sleep. The pathogenesis of SD and insomnia differs slightly, although the modeling methods used for SD and insomnia in animal experiments are identical and are established by the injection of p-chlorophenylalanine (PCPA) (6, 7). There is a lack of data on insomnia, and insomnia is an urgent clinical problem. Therefore, we believe that the key genes shown to be associated with SD in the present study are also applicable to insomnia.

Insomnia refers to a subjective experience in which patients are dissatisfied with the duration and quality of sleep, even though they have appropriate opportunities for sleep and access to a suitable sleep environment. The main clinical manifestations of insomnia include difficulty in falling asleep, dreaminess, easy awakening, and early awakening. More than 25% of adults worldwide suffer from these symptoms of insomnia, and 10% of these patients meet the diagnostic criteria for insomnia (8). The etiology of insomnia is complex, although its specific pathogenesis remains unclear. However, factors such as psychology, physiology, the environment, the use of certain medicines, life behavior, personality, spirit, and disease can induce insomnia. In a previous study, Kasper (9) found that an insufficient amount of sleep can cause low-grade inflammation and that this type of inflammation can further affect sleep. Inflammation and SD exert two-directional effects. Some studies have shown that insomnia is associated with abnormal sympathetic nervous system (SNS) and hypothalamic–pituitary–adrenal (HPA) axis functionality (10, 11). Previous research also found that 5-HT, dopamine, melatonin, and other indicators can be used for the routine detection of insomnia. Although previous researchers have identified some genes or indicators of insomnia, there is still no timely and effective means of diagnosing and monitoring insomnia by applying disease-related indicators. It would be highly beneficial to identify novel genes that can diagnose or predict insomnia. In addition, there is a significant relationship between insomnia and immunity, although there is only a limited body of evidence to support this association. Many studies have applied the use of CIBERSORT to analyze the characteristics of immune cells; however, this method has not been used to investigate insomnia.

We hypothesized that CIBERSORT could be used to identify the relationship between SD and the expression levels of key genes in the blood, tissues, or immune cells of patients with SD. In this study, data from four microarrays (GSE98564, GSE98565, GSE98566, and GSE82114) were merged into one comprehensive dataset to identify key genes in SD. Then, we analyzed the diagnostic value of the selected genes in SD using machine learning. Finally, we verified the genes identified by analysis of the Gene Expression Omnibus (GEO) dataset using qRT-PCR. Collectively, our findings provide new key genes related to the diagnosis of insomnia.

## 2 Materials and methods

### 2.1 Animal studies

Twelve healthy 6-week-old male Sprague Dawley rats weighing 200 ± 20 g were obtained from the Beijing Weitong Lihua Laboratory Animal Technology Co., Ltd., Beijing, China. The animals were reared in two separate cages (six per cage) in our animal husbandry center and were provided with standard sterile food and drinking water *ad libitum*. The rats were exposed to natural light with the room temperature between 22 and 26°C, and the humidity between 45 and 65%. The bedding was changed twice weekly to ensure that the rats lived in a well-regulated, quiet, and clean environment. After 7 days of adaptive rearing, the formal experiment was carried out. Eating, drinking, activity levels, and defecation were monitored daily. After all rats were confirmed to be healthy, they were assigned to either the control group (*n* = 6) or the insomnia model group (*n* = 6) using a random number table. The rats were intragastrically administered with 2 ml of saline once a day for 7 consecutive days. After 7 days of adaptive feeding, the control group was given an intraperitoneal injection of 2 ml, and the model group was given an intraperitoneal injection of PCPA (350 mg/kg) once a day for 3 consecutive days. After 7 days, the animals were weighed and euthanized using pentobarbital sodium. The hippocampus was removed and stored at −80°C for analysis. The study was approved by the ethics committee of the Hebei University of Chinese Medicine (Reference: DWLL2019024).

### 2.2 Quantitative real-time PCR (qRT-PCR)

Total RNA was extracted from the hippocampus, and 2 μg of total RNA was used for quantitative real-time fluorescence PCR detection. After amplification, the mRNA levels of each gene and an internal reference gene were calculated according to the Q = 2^−Δ*Cq*^ and RQ = = 2^−Δ*ΔCq*^ methods. The primers were as follows:

C2CD2L: forward (5′-CATGCCTGATGGCACAATCG-3′) and reverse (5′-CGGGAGGGGGAGTCTAGTTT-3′);

APOL3: forward (5′-GATACACACGGGAAGGACGG-3′) and reverse (5′-TGTGAGTCCAAGTGGAAATCCT-3′);

PKNOX1: forward (5′-TTGGCCGGATTCTCTTGCAT-3′) and reverse (5′-TCTGCGCCATCCTTGAAAGT-3′);

Beta-actin: forward (5′-GCAGGAGTACGATGAGTCCG-3′) and reverse (5′-GACAGGGACTGAAGGCTGTC-3′).

### 2.3 Microarray data

Four SD microarrays (GSE98564, GSE98565, GSE98566, and GSE82114) were downloaded from the NCBI Gene Expression Omnibus database (GEO; https://www.ncbi.nlm.nih.gov/geo/). GSE98564 included data relating to 128 specimens from patients with SD and 71 healthy specimens and is based on the GPL6244 [HuGene-1_0-st] Affymetrix Human Gene 1.0 ST Array [transcript (gene) version]. GSE98565 included data relating to 122 specimens from patients with SD and 71 healthy specimens and is based on the GPL6244 [HuGene-1_0-st] Affymetrix Human Gene 1.0 ST Array [transcript (gene) version]. GSE98566 included data from 92 specimens from patients with SD and 71 healthy specimens and is based on the GPL6244 [HuGene-1_0-st] Affymetrix Human Gene 1.0 ST Array [transcript (gene) version]. GSE82114 included data relating to 23 specimens from patients with SD and 26 healthy specimens and is based on GPL15331 Agilent-026817 University_Surrey_HumanSleep_44k_v1. We combined the four datasets using the R package SVA (12) after removing batch effects.

### 2.4 DEGs and the identification of differentially expressed genes

Next, we used the limma package (12) in the R software to identify differentially expressed genes (DEGs) and perform comparative analysis using the following conditions: |log2 Fold change (FC) |>0.1 and a false discovery rate (FDR) < 0.05. All these filter conditions needed to be met in order for DEGs to be defined.

### 2.5 Functional enrichment analyses

In this study, we used the “Cluster Profiler” package (12) in the R software to perform the Gene Ontology (GO) analysis and the Kyoto Encyclopedia of Genes and Genomes (KEGG) analysis based on the classification of high-risk and low-risk patients. For both GO and KEGG analyses, *p* < 0.05 was considered statistically significant.

### 2.6 The selection of candidate diagnostic markers

Next, we used two machine learning methods [Support Vector Machine Recursive Feature Elimination (SVM-RFE) and Least Absolute Shrinkage and Selection Operator (LASSO)] to predict SD status. LASSO (13) was applied in the “glmnet” package (12) in R to identify genes that are significantly related to SD samples compared to normal samples, while SVM used a recursive feature elimination (RFE) algorithm to select the key genes from a queue of metadata.

### 2.7 CIBERSORT analysis

Finally, we determined the immune response of 22 immune cells by applying the CIBERSORT (12) method and evaluated the correlation between the 22 different immune cells and the key genes that were identified earlier.

### 2.8 Statistical analysis

The *t*-test was used to compare gene expression levels between insomnia samples and normal samples. To test the classification effect of key genes on SD samples and normal samples, we generated receiver operating characteristic (ROC) curves and calculated the area under the curve (AUC) for each curve using the “proc” tool (12) in R. We used the R (version 4.2.1) and GraphPad Prism software for statistical analysis; ^*^p < 0.05.

## 3 Results

### 3.1 Determination of DEGs in sepsis

**Figure 1:**
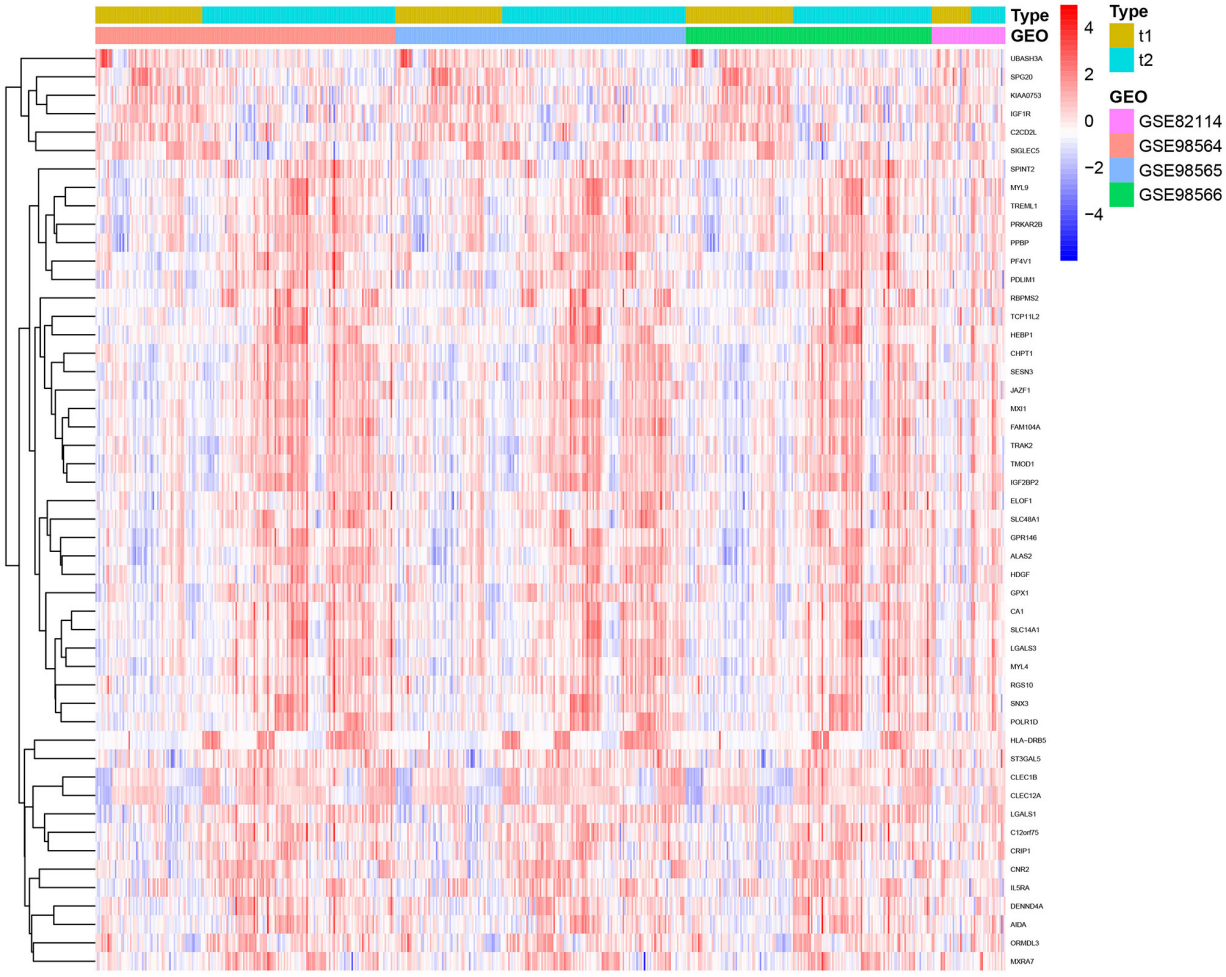
The identification of DEGs between insomnia and healthy subjects. t1 = control, t2 = SD.

After removing batch effects, a total of 50 genes were identified, out of which six genes were significantly upregulated and 44 genes were significantly downregulated (Figure 1).

### 3.2 Functional enrichment analyses

**Figure 2:**
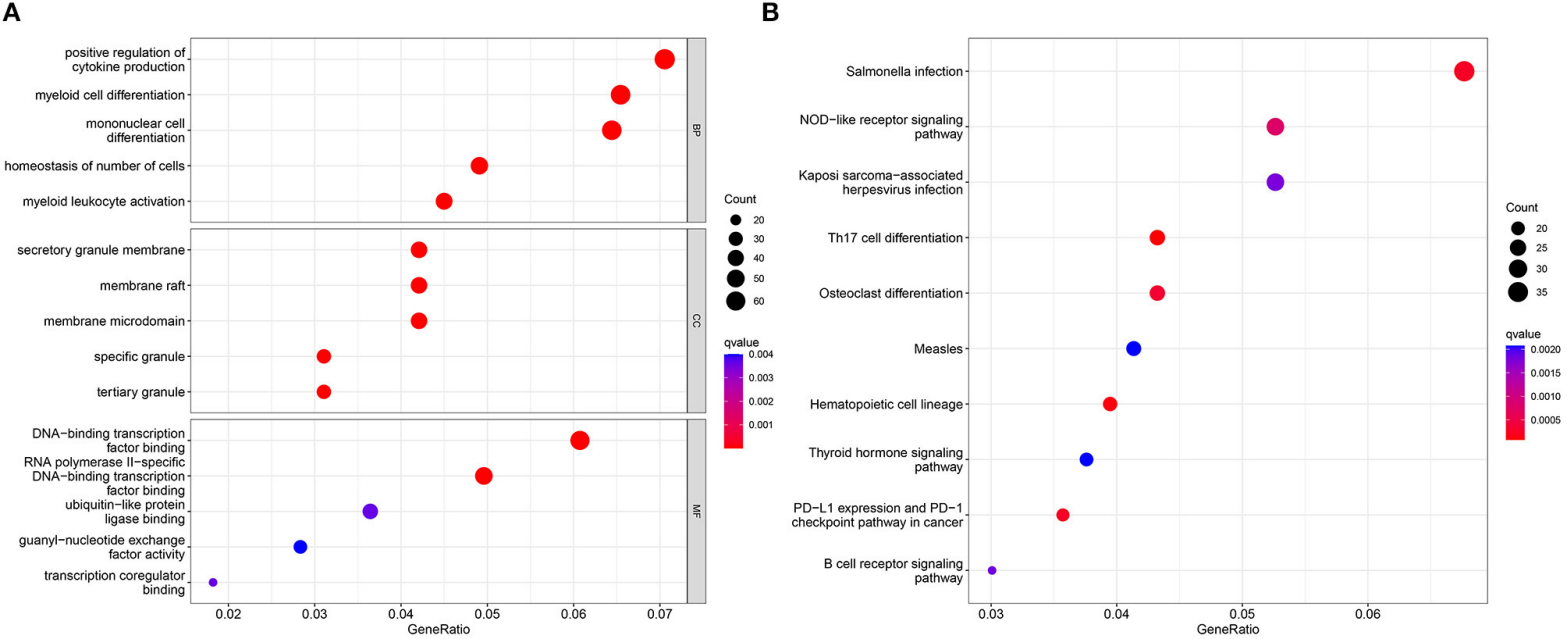
GO analysis **(A)** and KEGG analysis **(B)** for the 50 identified DEGs, as determined by the Cluster Profiler package.

GO analysis showed that the 50 DEGS identified in our analyses were mainly associated with the positive regulation of cytokine production, myeloid cell differentiation, and mononuclear cell differentiation (Figure 2A). KEGG analysis further showed that Salmonella infection and the NOD-like receptor signaling pathway were significantly associated with SD (Figure 2B).

### 3.3 The determination and verification of diagnosis markers

**Figure 3:**
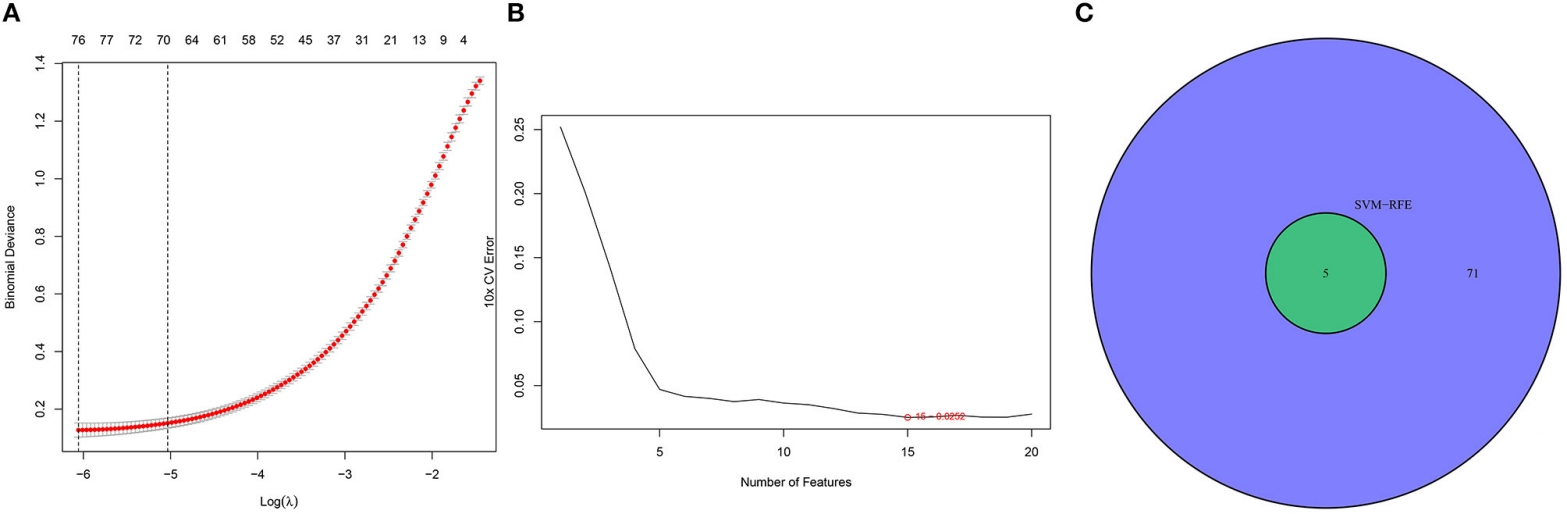
Selection of diagnosis marker candidates for insomnia: **(A)** the tuning of feature screening in the LASSO model; **(B)** biological marker screening *via* the SVM-RFE algorithm; and **(C)** a Venn diagram displaying three diagnosis biomarkers shared by the LASSO and SVM-RFE methods.

We identified DEGs by applying the LASSO regression algorithm and finally determined 76 variables as diagnostic markers of SD (Figure 3A). In addition, we identified 15 feature subsets in the DEGs by applying the SVM-RFE algorithm (Figure 3B). The five overlapping features between the two algorithms [C2 domain-containing protein 2-like (C2CD2L), Serine Peptidase Inhibitor Kunitz Type 2 (*SPINT2*), Apolipoprotein L3 (APOL3), PBX/knotted 1 homeobox 1v (PKNOX1), and alpha-2 macroglobulin (*A2M*)] were finally selected as the key genes involved in the progression of SD (Figure 3C).

### 3.4 The expression and diagnostic significance of *C2CD2L, SPINT2, APOL3, PKNOX1*, and *A2M* in SD

**Figure 4:**
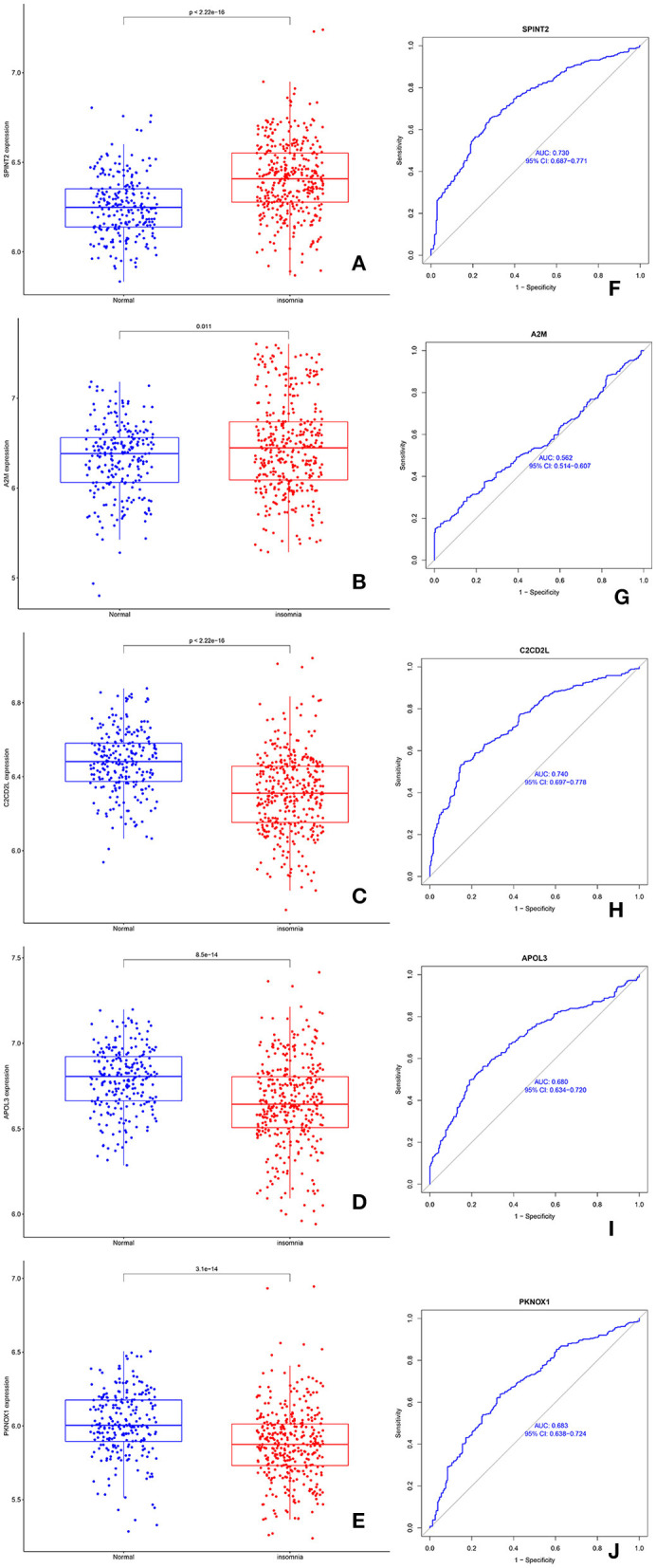
The expression and diagnostic significance of *C2CD2L, SPINT2, APOL3, PKNOX1*, and *A2M* in insomnia. **(A, B)** The expression levels of *SPINT2* and *A2M* were significantly downregulated in patients with insomnia. **(C–E)** The expression levels of *C2CD2L, APOL3*, and *PKNOX1* were significantly downregulated in patients with insomnia. **(F–J)** ROC curves for *SPINT*2, *A2M, C2CD2L, APOL3*, and *PKNOX1* in patients with insomnia.

Compared with normal samples, the expression levels of *SPINT2* and *A2M* were significantly upregulated in SD (Figures 4A, B). *C2CD2L, APOL3*, and *PKNOX1* were significantly downregulated in samples from patients with SD when compared to the control samples (Figures 4C–E). Next, we performed a ROC analysis for *C2CD2L, SPINT2, APOL3, PKNOX1*, and *A2M*. The analysis showed that the AUC for *SPINT2* (Figure 4F) was 0.730, the AUC for A2M (Figure 4G) was 0.562, the AUC for C2CD2L (Figure 4H) was 0.740, the AUC for APOL3 (Figure 4I) was 0.680, and the AUC for PKNOX1 (Figure 4J) was 0.683.

### 3.5 The expression levels of *C2CD2L, SPINT2, APOL3*, and *PKNOX* are associated with the extent of immunocyte infiltration

**Figure 5:**
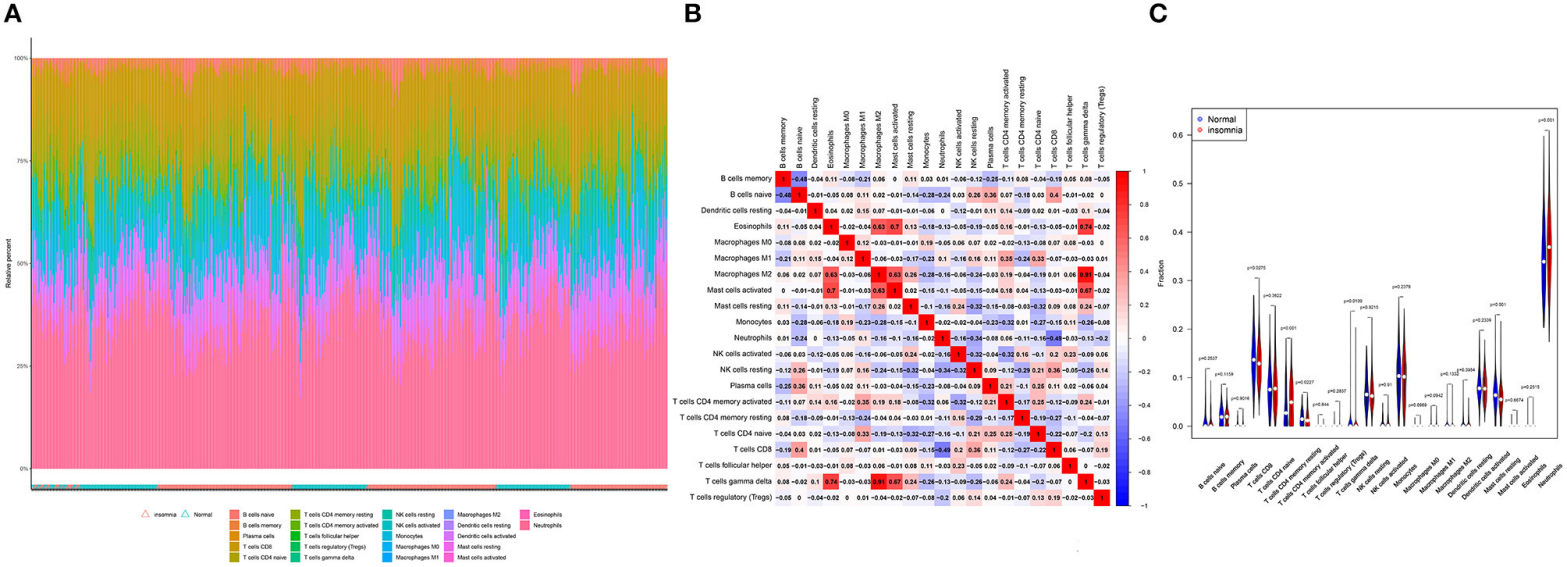
**(A, B)** The 22 immunocytes identified by the CIBERSORT software. **(C)** Diversities in the architecture of immunocytes between normal and insomnia samples.

**Figure 6:**
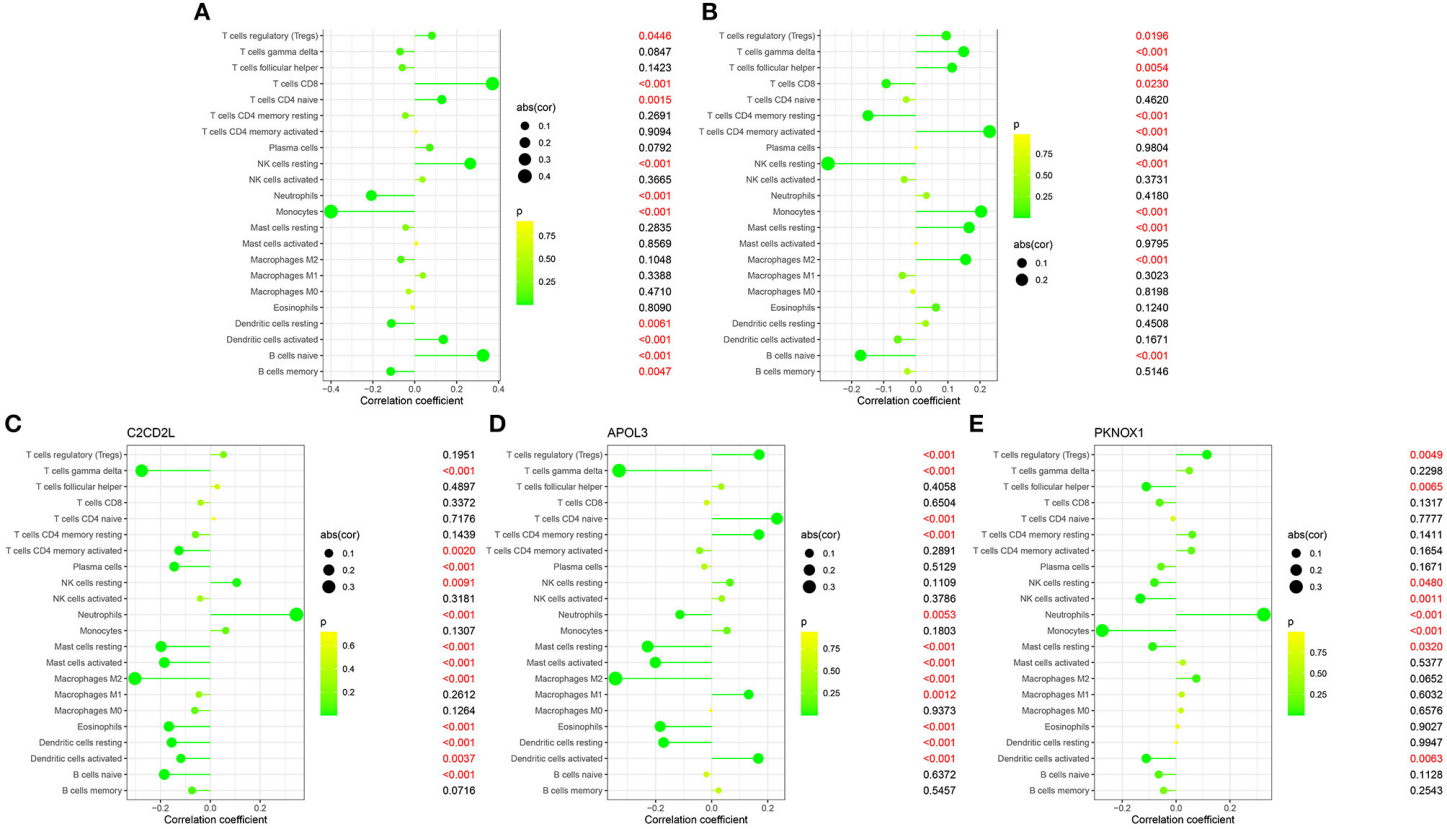
Correlations between *C2CD2L* **(A)**, *SPINT2* **(B)**, *APOL3* **(C)**, *PKNOX1* **(D)**, and *A2M* **(E)** and infiltrating immune cells in insomnia and normal samples.

We identified coefficients for *C2CD2L, SPINT2, APOL3, PKNOX1*, and A2M and the osmotic state of immune cells in normal and sleep-deprived samples, so that we could identify key relationships (Figures 5A, B). We identified a significant difference (*p* < 0.05) in CD8 T cells, resting CD4 T memory cells, memory-activated CD4 T cells, gamma delta T cells, resting mast cells, and neutrophils (Figure 5C). We further established a relationship between *C2CD2L, SPINT2, APOL3, PKNOX1*, and *A2M* expression and the degree of immune infiltration (Figures 6A–E). These key genes have clear associations with most of the 22 immune cells, thus suggesting that these genes may participate in SD by regulating multiple immune cells and therefore represent key biomarkers.

### 3.6 *In vivo* validation of the expression levels of three diagnostic genes

**Figure 7:**
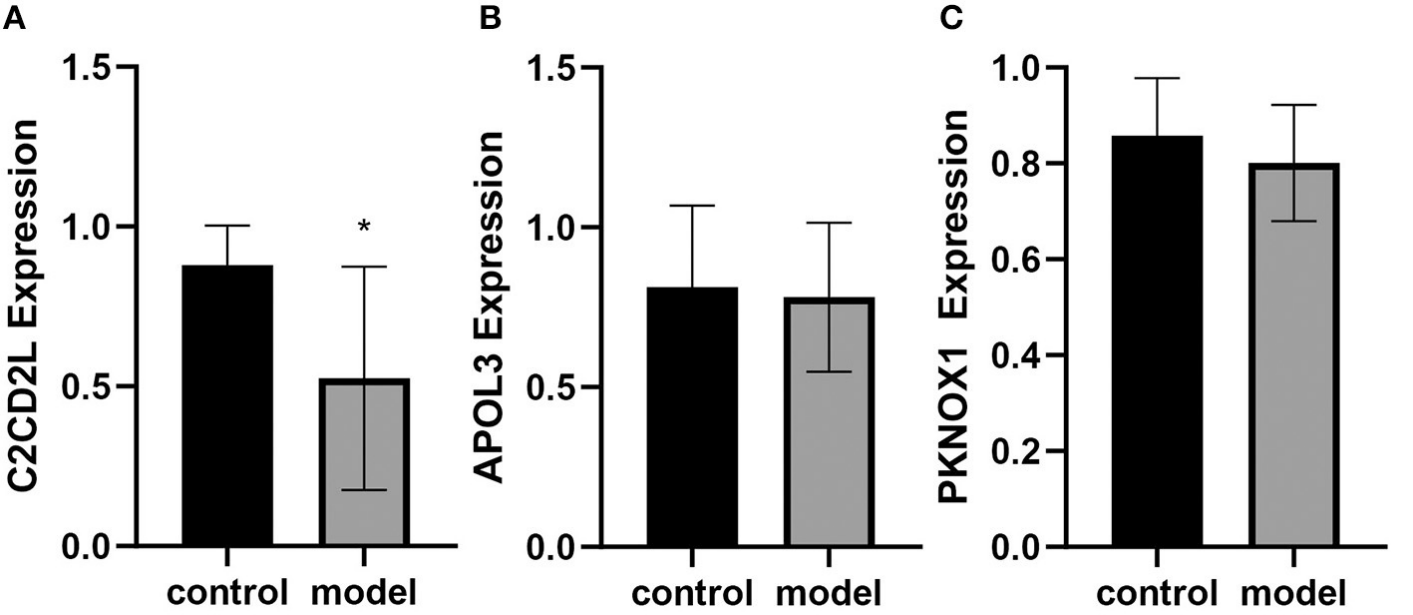
qRT-PCR was performed to determine the expression levels of *C2CD2L* **(A)**, *APOL3* **(B)**, and *PKNOX1* **(C)**, in insomnia samples (*n* = 6) and normal samples from a rat model. ^*^p < 0.05.

qRT-PCR was performed to determine the expression levels of *C2CD2L, APOL3*, and *PKNOX1* in rats with and without insomnia. The expression levels of *C2CD2L* in insomnia samples were significantly lower than those in normal samples, and *APOL3* and *PKNOX1* showed no significant changes in the two groups (Figures 7A–C).

## 4 Discussion

SD and insomnia have similarities in animal models and symptoms. Therefore, in this study, we consider both SD and insomnia. Insomnia and SD can be induced by the injection of PCPA, which is the most widely used drug for modeling. We also used this method to create a rat model. It can achieve the purpose of sleep deprivation by depleting 5-HT, NA, DA, and other brain serotonin. However, insomnia is also closely related to genetic factors, but PCPA modeling methods and genetic relationships were not significantly associated. One of the reasons is that there is a difference between the animal model and the clinic, which is a shortcoming of the animal model. At present, we can only evaluate the symptoms of sleep using objective scoring items. However, the timely diagnosis of insomnia is critical for patients to receive timely medication; there is an urgent need to develop an early diagnostic tool for use in clinical practice. Although melatonin, 5-HT, and DA are commonly used therapeutic targets for insomnia, these targets cannot be used to diagnose disease in a timely and effective manner. We need to develop other targets and genes that could be used to diagnose insomnia more effectively. To the best of our knowledge, this is the first study to investigate the use of GEO microarrays to identify biomarkers for patients with insomnia.

First, we identified the top 50 DEGs between SD samples and healthy samples; these DEGs were mainly associated with the positive regulation of cytokine production, myeloid cell differentiation, and mononuclear cell differentiation. The KEGG analysis further showed that Salmonella infection and the NOD-like receptor signaling pathway were also significantly associated with SD. Second, we identified five genes (*C2CD2L, SPINT2, APOL3, PKNOX1*, and *A2M*) using the LASSO regression algorithm and the SVM-RFE algorithm. Third, we predicted the expression of five genes and plotted ROC curves (*SPINT2* = 0.730, A2M = 0.562, C2CD2L = 0.740, APOL3 = 0.680, and PKNOX1 = 0.683). Fourth, we determined the relationship between C2CD2L, APOL3, and PKNOX1 and immune cells. Finally, we detected the mRNA expression of the rat hippocampus using qRT-PCR. The results show that the results for C2CD2L are meaningful and consistent with the predicted results. However, there was no significant difference in the expression of the other two genes between the normal group and the model group. These findings indicate that these mechanisms could be used to treat insomnia and may play a crucial role in the development of insomnia.

C2CD2L, also known as lipid transfer protein TMEM24, is a regulatory component in mammalian neurons. Abnormal levels of C2CD2L can lead to abnormal mitochondrial function, thus causing damage to ATP production and inducing abnormal metabolism (14, 15). Abnormal metabolism is one of the key manifestations of insomnia in patients. In a previous study, Zhang et al. (16) showed that patients with insomnia developed metabolic syndrome (hypertension, hyperglycemia, hyperlipidemia, and obesity) 1.41-, 1.29-, and 1.31-fold more often than normal subjects. However, at present, there is no significant evidence relating to the gene(s) that may cause metabolic abnormalities in patients with insomnia. In our future research, we will conduct related analysis and research on insomnia and metabolic genes and analyze the specific relationship between C2CD2L and insomnia. *SPINT2* is located at 19q13.1 and encodes a serine protease inhibitor that can regulate hepatocyte growth factor and inhibit fibrinolytic enzymes, insulin, plasma kallikrein, and other serine proteases. At present, SPINT2 is the focus of research related to cancer (17) and viral infections (18); there has been no research on the association between this enzyme and insomnia. APOL3 is a member of the APOL family of high-density lipoproteins and plays an important role in cholesterol transport. APOL is a host defense protein stimulated by IFN-g, has obvious bactericidal and protective effects on human cells, and exhibits a clear correlation with kidney disease, neurotransmission disorder, and cancer (19). PKNOX1 is known to play an important role in regulating immunity (20) and body metabolism (21) and can be used as a target cell to regulate the polarization of macrophages as well as sugar and lipid metabolism. *A2M* is located on chromosome 12; the protein uses bait-and-trap mechanisms to inhibit a wide range of proteases that are closely related to Alzheimer's disease (22).

Many lines of evidence indicate that the immune system may play a central role in the pathogenesis of SD (23, 24). Therefore, it is very important to evaluate the status of various immune cells from the perspective of immunity and then determine their individual components to reveal the causal relationship with SD and identify new targets for immunotherapy. In this study, the expression levels of *C2CD2L, SPINT2, APOL3, PKNOX1*, and *A2M* exhibited clear associations with most of the 22 immune cells tested, thus suggesting that these genes may participate in the development of insomnia by regulating multiple immune cells.

We have validated the level of *C2CD2L*, and the results were consistent with the predictions. Other genes were not identified or expressed differently between the two groups. However, it is still hopeful that *C2CD2L* could become a marker after a large number of experiments in the later stages. These results indicate that *C2CD2L* may represent a potential biomarker for the diagnosis of insomnia.

We acknowledge that this study has some limitations that need to be considered. First, we were unable to collect our own clinical data for verification. Second, we did not identify the specific relationships between genes and immune cells. Third, due to financial reasons, we were unable to validate all genes and validate the screened genes in other brain tissues.

## 5 Conclusion

Collectively, our data indicate that there are differences in the expression of the key gene (*C2CD2L*) between normal tissues and insomnia tissues. This gene may provide a new direction for the development of insomnia in the future.

## Data availability statement

The original contributions presented in the study are included in the article/supplementary material, further inquiries can be directed to the corresponding authors.

## Ethics statement

The study was approved by the Ethics Committee of Hebei University of Traditional Chinese Medicine (Reference: DWLL2019024). All experiments were conducted according to relevant guidelines and regulations.

## Author contributions

QW prepared the manuscript, performed data analysis, and was responsible for conceiving this research. JM and FC revised the manuscript. DL, YL, and ZL conceived and designed the research. YT, TG, and XL performed data analysis. All authors contributed to the article and approved the submitted version.
